# Our recommendations for acute management of COVID-19

**DOI:** 10.1186/s13054-020-02930-6

**Published:** 2020-05-08

**Authors:** Francesco Mojoli, Silvia Mongodi, Anita Orlando, Eric Arisi, Marco Pozzi, Luca Civardi, Guido Tavazzi, Fausto Baldanti, Raffaele Bruno, Giorgio Antonio Iotti, COVID-19 Pavia Crisis Unit, Carlo Marena, Monica Calvi, Giuseppina Grugnetti, Marco Maurelli, Alba Muzzi, Bruno Raffaele, Paolo Lago, Gianluigi Marseglia, Stefano Perlini, Alessandra Palo, Fausto Baldanti, Luigi Oltrona Visconti, Angelo Guido Corsico, Antonio Di Sabatino, Francesco Mojoli, Giorgio Iotti, Marco Benazzo, Nicora Carlo, Triarico Antonio, Petronella Vincenzo

**Affiliations:** 1 Department of Clinical-Surgical, Diagnostic and Paediatric Sciences, Unit of Anaesthesia and Intensive Care, University of Pavia, Pavia, Italy; 2 Anaesthesia and Intensive Care, San Matteo Hospital, Pavia, Italy; 3 Rianimazione I, Fondazione IRCCS Policlinico S. Matteo, DEA piano -1, Viale Golgi 19, 27100 Pavia, Italy; 4 Molecular Virology Unit, Microbiology and Virology Department, San Matteo Hospital, Pavia, Italy; 5 Infectious Diseases, San Matteo Hospital, Pavia, Italy

**Keywords:** ARDS, COVID-19, Acute respiratory failure, Lung ultrasound, Novel coronavirus

The 2019 coronavirus disease (COVID-19) epidemic is currently spreading worldwide; in particular, Italy and our region (Lombardy) have been facing the largest European outbreak since February 21st [1]. We here share our practical clinical management suggestions, derived from the direct experience of the first 200 patients with acute respiratory failure, of which 75 were finally admitted to intensive care unit (ICU) to undergo mechanical ventilation:

1. Suggestive clinical picture includes upper airways disease, with cough, fever and flu-like syndrome, evolving to dyspnoea after 2–10 days and presenting with bilateral chest infiltrates. Blood gas analysis initially shows moderate hypoxaemia, metabolic acidosis with/without respiratory compensation, normal lactates and increased anion gap; ketoacids are found in urinary sticks. Blood samples show high C-reactive protein, normal procalcitonin, increased lactate dehydrogenase, creatine phosphokinase, amylases, lipases and hyperglycaemia. A nasal swab for 2019 novel coronavirus is routinely performed in any upper/lower airways disease [2].
2. In suggestive clinical pictures, do not trust negative nasal swab: patients may have negative swabs but positive bronchoalveolar lavage. We suggest isolating suspected patients and using personal protective equipment as if positive; eventually, repeat the nasal swab and perform a bronchoalveolar lavage if possible. Rule out other potential aetiologies of acute respiratory failure with microbiological sampling, urinary antigens for *Streptococcus pneumoniae* and *Legionella pneumoniae*, serologies for atypical bacterial pneumonia and search of other respiratory viruses [3, 4].
3. Increase the number of ICU beds and prepare the wards to provide non-invasive ventilation: once the epidemic starts, patients requiring respiratory support increase exponentially. Organize a shared dataset of patients with non-invasive ventilation in the wards [5] and a daily round by an intensivist, to early identify the failing patients.
4. Set up specific therapy early in all positive/suspected patients: we systematically introduce hydroxychloroquine 200 mg tid and azithromycin 500 mg per day; prophylaxis is started with trimethoprim/sulphamethoxazole 2 vials/day if lymphopaenia, which is frequent. Discuss the potential interest of remdesivir 100 mg/day with infectious disease consultant. Consider introducing empiric antibiotic therapy, to be stopped in case of negative microbiological samples and negative procalcitonin [4].
5. Correct metabolic acidosis and hyperglycaemia early to decrease respiratory load with insulin infusion (target glycaemia < 150 mg/dL); if negative base excess persists at blood gas analysis, consider sodium bicarbonate infusion (starting dose 20 mEq/h).
6. Most of the patients respond to positive end-expiratory pressure: prefer helmet to mask for a continuous positive airway pressure trial to set moderate-high positive end-expiratory pressure (> 8 cmH_2_O), improve patient’s tolerance to prolong non-invasive support and prevent droplets’ spread [4]. For this same purpose, add a high-efficiency particulate air filter before the positive end-expiratory pressure valve or, better, connect the valve to wall gas aspiration [6–8].
7. Perform early intubation if poor response to continuous positive airway pressure in terms of oxygenation: do not trust patients’ relatively good respiratory mechanics and feeling of improved dyspnoea, since these patients may have relatively normal lung compliance and the only clinical sign of fatigue may be high respiratory rate. Connect ventilator expiratory valve to wall gas aspiration to limit droplets’ spread.
8. Once intubated, perform a closed system bronchoalveolar lavage to confirm diagnosis: minimize the use of fiberbronchoscopes to limit airways’ opening; we connect a bronchoalveolar lavage test tube to the closed aspiration system—mandatory in these patients—for deep bronchial sampling. Thereafter, repeat the sampling every 7 days for viral charge assessment and bacterial over-infection detection [4].
9. After intubation, evaluate basic lung mechanics: it usually shows a respiratory system compliance of 0.5–1 ml/cmH_2_O per kilogramme of predicted body weight with high recruitability at pressure-volume curve and normal resistances. These patients usually show good response to high positive end-expiratory pressure levels; calibrated oesophageal pressure may help its setting [9]. Consider neuromuscular blocking agents if deep sedation does not control the patient’s trigger and ventilation is not protective; perform daily a trial of neuromuscular blocking agents stop.
10. Prefer lung ultrasound to other imaging techniques: it is accurate in interstitial diseases and may show pathological signs before chest X-ray. A basic assessment helps deciding the ventilatory strategy: if diffuse loss of aeration, keep high positive end-expiratory pressure levels; if posterior consolidations, consider pronation. Lung ultrasound may also help in limiting traditional imaging, avoiding patients’ transportation to radiology department. It also allows a daily monitoring of clinical evolution, response to treatment and possible complications (pneumothorax, over-infections) [10–12].
11. Avoid positive fluid balance: perform fluid challenges and stop fluid resuscitation if no haemodynamic response; use vasoactive drugs instead to optimize tissue perfusion [4]. We accept moderate elevation of creatinine without urinary output impairment to improve the lung status.
12. Fever is a frequent issue, reaching values as high as 40 °C; we decided to treat it only if > 39 °C, if oxygenation is acceptable. Spontaneous defervescence can be the first sign of clinical improvement.
13. As soon as possible according to gas exchanges (PaO_2_/FiO_2_ > 150 with FiO2 < 50%) and lung ultrasound score (≤ 12), start assisted ventilation with a sigh while maintaining moderate to high positive end-expiratory pressure to prevent derecruitment. Regularly check patient’s respiratory drive (P0.1), tidal volume and plateau pressure to keep ventilation safe. Dexmedetomidine may help in the weaning phase.
14. In patients having received prolonged sedation, we frequently observed prolonged awakening with altered respiratory drive and difficult patient-ventilator interaction; if no prompt awakening is observed, perform early tracheostomy to accelerate the weaning and discharge from ICU. The number of patients requiring intensive care rapidly increases; therefore, rapid discharge is mandatory.
15. Hyperinflammatory status increases the risk of thrombosis and pulmonary embolism; check for thrombotic complications systematically, mainly in correspondence of central lines [13, 14].
16. Communication with families is difficult since patients’ relatives are frequently in quarantine and access to the hospital is limited; moreover, while wearing personal protective equipment, physicians’ possibility to answer to relatives’ phone call is limited. Consider identifying each day one person in charge of phone calls to daily update relatives on clinical conditions [15].

We hope sharing our experience while facing the Italian outbreak of 2019 novel coronavirus may help other Units eventually facing the same threat in the future.

## Data Availability

Not applicable

## Abbreviations

SARS-CoV-2: Severe acute respiratory syndrome coronavirus 2
ICU: Intensive care unit
